# Hormesis effects of phenol on growth and cellular metabolites of *Chlorella* sp. under different nutritional conditions using response surface methodology

**DOI:** 10.1007/s11356-023-26249-1

**Published:** 2023-03-17

**Authors:** Mohamed Gomaa, Eman H. El-Naeb, Awatief F. Hifney, Mahmoud S. Adam, Mustafa A. Fawzy

**Affiliations:** 1 Botany & Microbiology Department, Faculty of Science, Assiut University, Assiut, 71516 Egypt; 2 Biology Department, Faculty of Science, Taif University, 21974 Taif, Kingdom of Saudi Arabia

**Keywords:** Hormesis, Response surface methodology, Phenol toxicity, Mixotrophic, Antioxidant enzymes, *Chlorella*

## Abstract

The present study investigated the effects of different phenol concentrations (200 – 1000 mg L^−1^) towards *Chlorella* sp. under different culture conditions (light *vs.* dark) and NaNO_3_ concentrations (0 – 0.1 g L^−1^) using central composite design. Phenol induced hormesis effects on the algal growth and cellular metabolites. Nitrate was identified as a crucial factor for promoting the uptake of phenol by *Chlorella* cells, while light was a limiting factor for growth, but the phyco-toxicity of phenol was decreased in the dark. The pigment contents were generally increased in the treated cells to protect against the oxidative phenol stress. The incorporation of 200 mg L^−1^ phenol and 0.05 g L^−1^ NaNO_3_ to the illuminated cells markedly promoted biomass and lipid contents to 0.22 g L^−1^ and 26.26% w/w, which was 44 and 112% higher than the phenol-less control, respectively. Under the same conditions, the increase of phenol concentration to 600 mg L^−1^, the protein contents were increased to 18.59% w/w. Conversely, the algal cells were able to accumulate more than 60% w/w of soluble carbohydrates under dark conditions at 600 mg L^−1^ of phenol. Nitrate replete conditions stimulated lipid accumulation at the expense of protein biosynthesis. Furthermore, most of the treatments showed an increase of H_2_O_2_ and malonaldehyde contents, especially for the illuminated cells. However, catalase activity tended to increase under dark conditions, especially at low phenol and nitrate concentrations. This study is valuable in indicating the effects of phenol on microalgae by exploiting response surface methodology, which can be applied as a powerful tool in growth monitoring and toxicity assessment.

**Supplementary Information:**

The online version contains supplementary material available at 10.1007/s11356-023-26249-1.

## Introduction

Phenol and its derived compounds are ubiquitous pollutants, which enter the environment through several natural and anthropogenic sources. Natural phenolic pollution is related to the incomplete biodegradation of dead biota. In industry, phenol is used as a common ingredient in many products such as disinfectants, soaps, paints, perfumes, and varnish removers as well as various pharmaceutical products (Anku et al. 2017). Therefore, the effluents from petroleum processing plants, oil refineries, plastic, paper and pulp, pharmaceutical and agrochemical industries represent the main sources of anthropogenic phenol contamination in the environment (Mohammadi et al. 2015).

Phenol can induce negative short- and long-term effects on all living organisms (Mahugo-Santana et al. 2010). It can also cause toxic effects to different biota even at concentrations as low as 5 mg L^−1^ (Surkatti and Al-Zuhair 2018). Therefore, phenol and its related compounds were generally listed as priority pollutants based on the United States Environmental Protection Agency (USEPA) and the European Union (EU) (Anku et al. 2017).

Microalgae are essential primary producers in the aquatic environments, which are characterized by small cell size and large surface area. The exposure of microalgal cells to organic pollutants such as phenol can markedly reduce or inhibit their net carbon fixation, growth rate and biosynthesis of some metabolites. For instance, growth, nitrate reductase activity, chlorophylls (*a* and *b*), total protein, and carbohydrate levels were markedly reduced in the chlorophycean algae *Chlorella vulgaris* and *Scenedesmus bijugatus* under phenol stress (Megharaj et al. 1992). The phyco-toxicity of phenol may be attributed to its cellular penetration and hydrophobic interactions with the cell membrane and membrane-bound organelles, leading to disruption of their structure and function (Scragg 2006). Accordingly, several ultrastructural alterations were observed after exposing microalgal cells to phenol such as the disappearance, or shrinkage, of nucleolus, vesiculation or enlargement of vacuoles, cell wall thickening, and damaging of chloroplast lamellae (Megharaj et al. 1992; Duan et al. 2017). Furthermore, phenol induces the formation of free radicals and reactive oxygen species such as superoxide radicals and hydrogen peroxide as well as it can donate electrons to the oxidized biomolecules, due to its high reactivity (Michałowicz and Duda 2007).

On the other hand, several microalgae have been reported to mineralize phenol completely under sub-lethal concentrations (El-Gendy and Nassar 2021). The biodegradation of phenol by microalgae and its utilization as a carbon source for metabolism can increase the biomass productivity with simultaneous accumulation of certain metabolites (Gomaa et al. 2022). For instance, algal cells tend to accumulate lipids under phenol stress, which may be ascribed as a detoxification mechanism (Das et al. 2016; Cho et al. 2016). Accordingly, the simultaneous treatment of wastewater, that might be contaminated by high concentration of phenol, and biomass utilization as a biodiesel feedstock is an environmentally sustainable process.

In the presence of organic compounds such as phenol, microalgae can switch their metabolism from autotrophy to mixotrophy. During mixotrophic nutrition, algae can perform photosynthesis in the presence of light and utilize organic compounds simultaneously (Gomaa and Yousef 2020; Fawzy and Gomaa 2020). Thus, both autotrophic and heterotrophic mechanisms are present in the mixotrophic mode of growth (Gomaa and Ali 2021). The biodegradation of phenol by microalgae was reported to occur under mixotrophic or heterotrophic conditions (Semple and Cain 1996; Das et al. 2016; Gomaa et al. 2022). Several microalgae have been reported to use phenol as an energy and carbon source such as *Chlorella* sp., *Scenedesmus obliquus*, *Spirulina* sp. (Klekner and Kosaric 1992), *Chlorella pyrenoidosa* (Das et al. 2015), *Isochrysis galbana* (Wang et al. 2019), *Chlorella vulgaris*, *Acutodesmus obliquus* and *Monoraphidium braunii* (Lindner and Pleissner 2021), and *Chlorella* sp. and *Tetradesmus obliquus* (Fawzy et al. 2022). The phyco-toxicity of phenol have been investigated against several algae including marine microalgae (*Dunaliella salina, Platymonas subcordiformis, Phaeodactylum tricornutum, skeletonema costatum,* and *Lingulodinium polyedrum*) (Martins et al. 2015; Cho et al. 2016; Duan et al. 2017), freshwater microalgae (*Scenedesmus abundans*) (Fawzy and Alharthi 2021), and soil microalgae (*Chlorella vulgaris* and *Scenedesmus bijugatus*) (Megharaj et al. 1992).

Nitrate as a macro-element plays a significant role in the microalgal growth and metabolism. Additionally, nitrate and phenol are common co-occurring pollutants in several industrial wastewaters (Sarfaraz et al. 2004). Therefore, it is important to identify the main effects and interactions between phenol and nitrate on the microalgal growth and metabolism. Furthermore, the consumption of phenol by microalgae and mode of nutrition is mainly influenced by the existence of light as an energy source for the illuminated cells. To identify the main interactions between the aforementioned factors, response surface methodology as a powerful statistical tool can be used. This method has been employed to investigate the effect of joint toxicity of different pharmaceutical contaminants on microalgae (Gomaa et al. 2021). To the best of our knowledge, no attempts have been carried out to investigate the positive and negative effects of phenol towards microalgae under different trophic conditions such as nitrate concentration and light.

The aim of the present study was set to investigate the positive and negative effects of phenol to the green microalga *Chlorella* sp. under different nitrate concentrations in the presence or absence of light. The effects of these factors were compared in response of phenol removal, growth inhibition, pigments (Chl. a and carotenoids), proteins, lipids, carbohydrates, stress biomarkers (H_2_O_2_ and malonaldehyde), and antioxidant enzymes (catalase and ascorbate peroxidase) using response surface methodology.

## Materials and methods

### Algal isolate and growth conditions

*Chlorella* sp. (Chlorophyta) was isolated from polluted water sample collected from Assiut, Egypt (N 27°10′11", E 31° 9′ 27"). Microalgal isolation, purification and cultivation were performed using Bold’s Basal medium (BBM). The composition of BBM was (per one liter of distilled water): 250 mg NaNO_3_, 75 mg MgSO_4._7H_2_O, 25 mg NaCl, 75 mg K_2_HPO_4_, 175 mg KH_2_PO_4_, 25 mg CaCl_2_.2H_2_O, 11.42 mg H_3_BO_3_, 8.82 mg ZnSO_4_.7H_2_O, 1.44 mg MnCl_2_.4H_2_O, 0.71 mg MoO_3_, 1.57 mg CuSO_4._5H_2_O, 0.49 mg Co(NO_3_)_2_.6H_2_O, 50 mg Na_2_EDTA, 31 mg KOH, 4.98 mg FeSO_4_.7H_2_O, and 1.84 mg H_2_SO_4_ (Bischoff and Bold 1963). The isolated *Chlorella* sp. cells are solitary, oval to ellipsoidal, 5 – 8.3 × 6 – 8.5, and contains single cup-shaped chloroplast (Fig. S1). Algal cultivation was carried out in 1000 mL glass bottles containing 750 mL BBM medium under continuous illumination (48.4 µmol m^−2^ s^−1^) and gassed with sterile air provided by air pumps (0.5 vvm) at 25 °C.

### Experimental conditions

In 100 mL Erlenmeyer conical flasks, 50 mL of BBM were inoculated with 7-days old *Chlorella* sp. cells to give a constant final optical density of 0.1 at 750 nm. Different concentrations of phenol (200, 400, 600, 800 and 1000 mg L^−1^) were investigated with different sodium nitrate concentrations (0, 0.025, 0.05, 0.075 and 0.1 g L^−1^) and culture conditions (mixotrophic vs. heterotrophic). To induce mixotrophic growth, the algal cultures were incubated under continuous illumination (48.4 µmol m^−2^ s^−1^) and gassed with sterile air provided by air pumps. While the heterotrophic growth was performed in the dark and static conditions. The effects of phenol, sodium nitrate and mode of nutrition were estimated based on central composite design (CCD) and response surface methodology. The design consisted of 26 runs including five replicates at the center point to estimate the experimental error. Each treatment was consisted of triplicate flasks and cultivations were proceeded at 25 °C for 4 days. Phenol-free cultures grown in BBM under the same conditions were referred to as negative controls (Table 1).

**Table 1:** Effect of different nitrate concentrations under light and dark condition on the growth and biochemical composition of *Chlorella* cells grown in phenol-free cultures

Factors			Responses									
Controls	NaNO_3_ (g L^−1^)	Culture condition	Biomass (g L^−1^)	Chl a (mg g^−1^)	Carotenoids (mg g^−1^)	Carbohydrate (%)	Lipid (%)	Protein (%)	CAT (Umg^−1^)	APX (Umg^−1^)	H_2_O_2_ (µmol/g)	MDA (µmol/g)
C1	0	Light	0.168	6.31	7.01	17.52	18.98	8.52	5.29	1.22	9.47	23.97
C2	0.025	Light	0.166	4.47	6.41	21.34	11.63	7.29	6.00	3.38	12.83	63.42
C3	0.05	Light	0.153	7.65	8.61	19.26	12.38	10.71	4.88	2.13	8.69	14.29
C4	0.075	Light	0.172	5.74	6.30	20.62	12.31	10.75	7.82	2.02	3.88	39.82
C5	0.1	Light	0.164	9.97	7.25	17.97	13.56	13.11	3.30	3.30	17.83	75.03
C6	0	Dark	0.133	5.21	6.65	24.89	22.73	5.56	6.59	3.66	19.04	25.87
C7	0.025	Dark	0.123	3.23	5.06	28.69	18.71	6.81	8.78	5.85	12.94	42.02
C8	0.05	Dark	0.128	7.73	8.76	58.21	17.82	7.71	9.76	6.10	14.58	7.65
C9	0.075	Dark	0.131	6.43	6.81	45.10	18.40	8.31	7.75	5.17	15.25	32.68
C10	0.1	Dark	0.132	8.19	8.36	51.03	15.89	7.93	10.78	5.39	10.09	40.61

CAT: catalase; APX: ascorbate peroxidase; MDA: malonaldehyde

The CCD was based on the following general equation (Gomaa and Yousef 2020; Gomaa and Ali 2021):

$$Y={\beta }_{0}+ \sum {\beta }_{i}{X}_{i}+ \sum {\beta }_{ii}{X}_{i}^{2}+\sum {\beta }_{ij}{X}_{i}{X}_{j}$$

where *Y* is the predicted response, *β*_*0*_,* β*_*i*_, *β*_*ii*_ and *β*_*ij*_ are the model intercept, linear, quadratic, and interaction coefficients, respectively. While *Xi* and *X*_*j*_ are the independent variables.

## Analytical methods

### Determination of algal growth

Algal growth was monitored by optical density (OD) at 750 nm using a UV–vis spectrophotometer (Model Unico UV − 2100, USA) (Gomaa and Ali 2021). The optical density values were converted into biomass concentration (g L^−1^) using the equation obtained by linear regression analysis of the relationship between the algal OD and its dry weight (DW). Dry cell weights were determined gravimetrically after collecting a series of algal cells with different OD by centrifugation (4800 g, 15 min) and oven drying (70 °C) till a constant weight (Fawzy and Gomaa 2020).

### Determination of photosynthetic pigments

Algal cells from 10 mL cultures were collected by centrifugation (4800 g, 15 min). The collected pellet was extracted using 80% (v/v) methanol in a water bath (70 °C) for 10 min. The extracted pigments were separated by centrifugation (4800 g, 15 min) and the absorbance of the supernatant was measured spectrophotometrically at 452, 644 and 663 nm against the methanol blank (Wellburn 1994). The concentrations of chlorophyll a (Chl. a), a7 to the following equations:

$$\mathrm{Chl}.\;\mathrm a\;(\mathrm{\mu g}\;\mathrm{mL}^{-1})\;=\;(10.3\times{\mathrm A}_{663})\;-\;(0.918\times{\mathrm A}_{644})$$

$$\mathrm{Carotenoids}\;{{\mu}\mathrm{g}}\;{\mathrm{mL}^{-1}})\;=\;4.2\;\times{\mathrm A}_{452}\;-\;(0.0264\times{\mathrm{Chl}}.\;\mathrm{ a}+0.426\times\mathrm{Chl}.\;\mathrm {b})$$

where A represents the absorbance at the corresponding wavelength.

### Determination of residual phenol concentration in the algal medium

One milliliter of the algal culture was withdrawn at the beginning and the end of the experiment, and the algal cells were removed by centrifugation (4800 g, 15 min). Residual phenol concentration was determined in the supernatant by the 4-aminoantipyrine method (Emerson 1943). Each sample (1 mL) was mixed with Na_2_CO_3_/NaHCO_3_ buffer (60 mM: 40 mM, pH 10.0, 1 mL), Na_2_CO_3_ (0.1 M, 1 mL), 4 − amino − antipyrine (0.06% w/v, 1 mL), and potassium ferricyanide (0.24% w/v), respectively. The developed red color was measured spectrophotometrically at 510 nm against a suitable blank without phenol.

### Estimation of cellular lipid contents

Algal lipid content was measured by sulfo-phospho-vanillin method (Mishra et al. 2014). Algal cells were collected by centrifugation (4800 g, 15 min), and resuspended in a known volume of distilled water. An aliquot of each algal sample (100 µL) was mixed with concentric sulfuric acid (2 mL) then placed in a boiling water bath for 10 min. After cooling, 5 mL of phosphovanillin reagent was added (0.6 g vanillin dissolved in 10 mL of absolute ethanol and completed to 100 mL with distilled water, then 400 mL of concentric phosphoric acid), and the mixture was kept in a 37 °C water bath for 15 min. The absorbance was measured spectrophotometrically at 530 nm against a suitable blank. Sunflower oil was used as a standard.

### Determination of cellular carbohydrate contents

The microalgal soluble carbohydrate contents were estimated at the end of the cultivation period by the UV-sulfuric acid method (Albalasmeh et al. 2013). The centrifuged algal cells were suspended in 2 mL distilled water and kept in a boiling water bath for 2 h to extract soluble sugars. The clear supernatant (0.5 mL) after centrifugation (4800 g, 15 min) was mixed with concentric sulfuric acid (1.5 mL). The absorbance was measured spectrophotometrically at 315 nm against a suitable blank and glucose was used as a standard.

### Determination of total protein contents

The algal protein contents were estimated by Lowery method (Lowry et al. 1951). The centrifuged algal cells were hydrolyzed by NaOH (1N) in a boiling water bath for 2 h. After centrifugation, 1 mL of the clear supernatant was mixed with 1 mL of Lowry reagent followed by 200 µL of Folin–Ciocalteau reagent. The absorbance was measured at 750 nm and bovine serum albumin was used as a standard.

### Determination of stress biomarkers

The collected algal cells were homogenized in 0.1% (w/v) trichloroacetic acid (TCA) and centrifuged to remove the cell residues. The supernatant was used for the determination of hydrogen peroxide (H_2_O_2_) and malonaldehyde (MDA) contents.

For the determination of H_2_O_2_, the supernatant (1 mL) was mixed with 1.5 mL phosphate buffer (50 mM, pH 7) and 1 mL potassium iodide (1 M). The developed yellow color was estimated at 390 nm using a spectrophotometer.

Malonaldehyde (MDA) concentration was measured as a biomarker for lipid peroxidation (Hodges et al. 1999). One milliliter of the supernatant was mixed with 5% TCA (2 mL) containing 0.5% (w/v) thiobarbituric acid (TBA), and the reaction was incubated at 95 ℃ in a water bath for 45 min. After centrifugation, the absorbance was measured at 450, 532 and 600 nm using a spectrophotometer. The MDA content was expressed as µmol g^−1^ DW. using the following equation:

$$\mathrm{MDA }\left(\mathrm{\mu mol }{\mathrm{g}}^{-1}\mathrm{ DW}\right)=\frac{\left[6.45\times \left({\mathrm{A}}_{532}-{\mathrm{A}}_{600}\right)\right]-\left(0.56\times {\mathrm{A}}_{450}\right)}{\mathrm{Dry \space weight}\left(\mathrm{g}\right)}$$

### Estimation of antioxidant enzymes’ activities

At the end of the experiment (day 4), the microalgal cells were collected by centrifugation (4800 g, 15 min) and resuspended in 3.0 mL phosphate buffer (50 mM, pH 7) and homogenized by sonication at 4 °C. After centrifugation, the supernatant was used for the determination of catalase (CAT; EC 1.11.1.6) and ascorbate peroxidase (APX; EC 1.11.1.11) activities.

CAT activity was determined by measuring the reduction of hydrogen peroxide H_2_O_2_ absorbance at 240 nm for 30 s using a spectrophotometer (Matsumura et al. 2002). The reaction mixture contained 2.8 mL phosphate buffer (50 mM, pH 7), 100 µL H_2_O_2_ (45 mM) and 100 µL of the algal extract.

APX activity was estimated by monitoring the decrease of ascorbate absorbance at 290 nm (Nakano and Asada 1987). The reaction solution contained 1.8 mL phosphate buffer (50 mM, pH 7) mixed with 20 µL of ascorbic acid (0.5 mM), 0.1 mM Na_2_-EDTA, 100 µL H_2_O_2_ (0.1 mM), and 100 µL of microalgal extract.

### Statistical analysis

All the analyses were performed using triplicate experiments. The statistical analysis for the central composite design (CCD) was performed using Design Expert 12.0 (Stat-Ease, Inc., Minneapolis, USA). The experimental data were analyzed by multiple regression analysis and one-way analysis of variance (ANOVA). Significant values were determined at p < 0.05.

## Results

### Effect of the investigated factors on the phenol removal (%) by *Chlorella* cells

The effects of phenol, NaNO_3_ and mode of nutrition (mixotrophic *vs.* heterotrophic) on *Chlorella* cells were estimated using a central composite design (CCD) and response surface methodology. After 96 h of exposure, the responses of phenol removal, algal growth inhibition, Chl. a, carotenoids, carbohydrates, lipids, proteins, CAT, APX, H_2_O_2_ and MDA were estimated as percentages in relation to the respective phenol-less controls (Table 1).

**Fig. 1:**
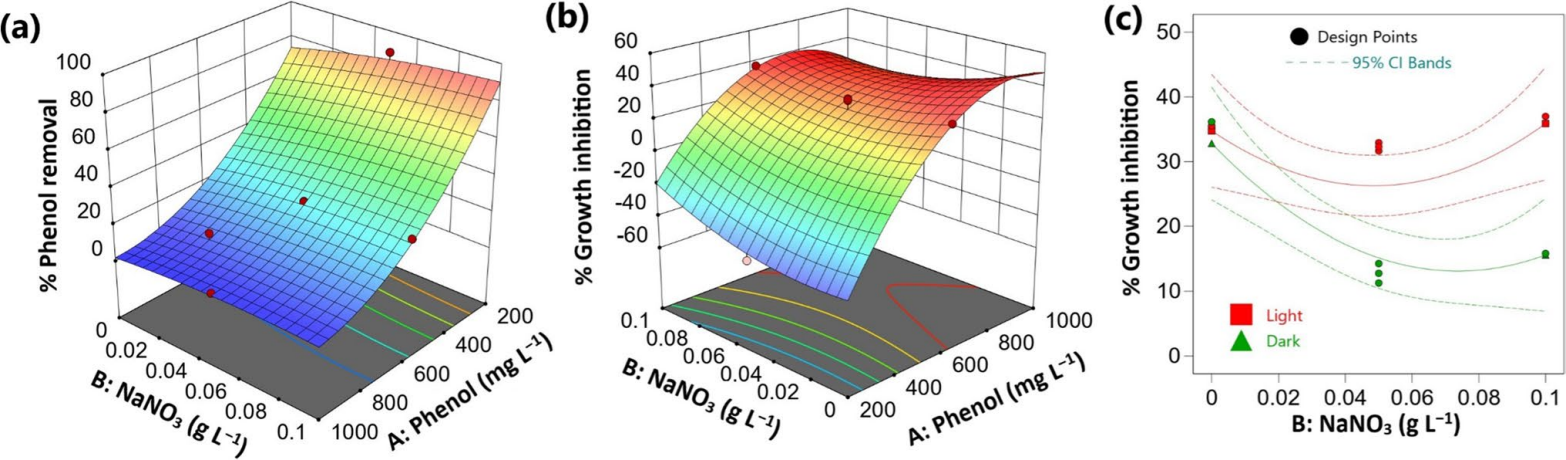
Effect of process variables on phenol removal and growth inhibition of *Chlorella* sp. (**a**), (**b**) 3-D response surface plots under light conditions and (**c**) interaction plot at 600 mg L^−1^ of phenol. CI: confidence interval

The initial concentration of phenol and sodium nitrate in the culture medium exhibited significant effects on the phenol removal (%) by *Chlorella* cells. The microalgal ability to consume phenol was decreased with increasing its initial concentration in the culture medium (Fig. 1a). However, the opposite trend was observed by increasing nitrate concentration, thus NaNO_3_ concentration exhibited positive effects on phenol removal (Table S1, Fig. 1a). Accordingly, the lowest (~ 7.5%) and highest (~ 88.5%) phenol removal were observed respectively at 1000 mg L^−1^ and 200 mg L^−1^ of phenol, in the presence of 0.05 g L^−1^ of NaNO_3_ (Table 2). Furthermore, these results were similar under mixotrophic and heterotrophic conditions. In other words, light exhibited non-significant effects on the phenol removal by *Chlorella* cells. ANOVA analysis also showed that the investigated factors had non-significant mutual interactions on the phenol removal (%) (Table S1).

**Table 2:** Effect of different of different phenol and sodium nitrate concentrations on phenol removal, growth and pigment contents of *Chlorella* cells under light and dark condition

Factors				Responses						
No	Phenol (mg L^−1^)	NaNO_3_ (g L^−1^)	Culture condition	Phenol removal (%)	Biomass (g L^−1^)	Growth inhibition (%)^a^	Chl a (mg g^−1^)	Chl a increase (%)^b^	Carotenoids (mg g^−1^)	Carotenoids increase (%)^b^
1	200	0.05	Light	88.52	0.221	-44.20	6.42	-16.03	5.13	-40.37
2	400	0.025	Light	45.69	0.173	-2.56	6.63	74.60	6.35	16.78
3	400	0.075	Light	49.57	0.159	4.05	5.57	-16.84	5.21	-29.20
4	600	0	Light	16.61	0.111	35.52	7.78	23.24	6.80	-3.03
5	600	0.05	Light	29.23	0.104	32.29	11.43	49.36	17.10	98.53
6	600	0.1	Light	32.72	0.103	36.98	6.06	-39.22	8.94	23.28
7	800	0.025	Light	13.55	0.106	37.04	8.28	60.69	8.32	12.71
8	800	0.075	Light	8.42	0.115	30.82	6.18	29.07	7.96	51.39
9	1000	0.05	Light	7.49	0.114	25.55	2.93	-61.64	8.03	-6.75
10	200	0.05	Dark	88.52	0.166	-43.78	3.35	-52.99	4.92	-39.18
11	400	0.025	Dark	51.51	0.141	-6.33	6.55	109.82	5.33	12.39
12	400	0.075	Dark	51.51	0.166	-6.84	4.94	-34.61	4.30	-46.14
13	600	0	Dark	17.23	0.084	36.15	6.69	28.28	7.73	16.17
14	600	0.05	Dark	32.13	0.111	12.78	9.53	33.55	14.19	75.51
15	600	0.1	Dark	45.93	0.111	15.85	4.02	-50.85	5.86	-29.94
16	800	0.025	Dark	15.55	0.098	26.04	6.25	86.81	6.59	22.55
17	800	0.075	Dark	18.41	0.104	15.56	4.42	-16.73	7.20	28.15
18	1000	0.05	Dark	7.86	0.099	22.56	4.33	-57.17	10.18	-11.25

^**a**^ % growth inhibition = [(Algal biomass in the control – Algal biomass in the treatment)/ Algal biomass in the control] × 100

^**b**^ % Increase = [(Treatment value – Control value)/ control value] × 100, values were calculated in relation to the corresponding control values listed in Table S1

### Effect of the investigated factors on the algal growth

The algal growth in the phenol treated cultures were compared to the corresponding control and the results were expressed as growth inhibition (%) in relation to the control (Table 1,2). Increasing phenol concentration showed negative effects on the algal growth, thus the algal growth was significantly inhibited by increasing initial phenol concentrations (Table S1, Fig. 1b). However, it is worth to mention here that phenol at low concentrations (200 and 400 mg L^−1^) had stimulatory effects on the algal growth, therefore the growth in these treatments was higher than the control cultures (Table 2). At 200 mg L^−1^ phenol and 0.05 g L^−1^ NaNO_3_, the algal concentration reached 0.221 g L^−1^ in the presence of light and 0.166 g L^−1^ under dark, which was more than 40% higher than the respective phenol-less controls (Table 2). Accordingly, the mode of nutrition exhibited highly significant negative effects on the growth inhibition (%); the algal growth inhibitory effect of phenol was higher under heterotrophic conditions than the mixotrophic conditions (Table S1, Fig. 1c). On the other hand, variations in NaNO_3_ concentration showed non-significant effects on the algal growth inhibition (%) in linear terms but exhibited significant negative mutual interaction with the mode of nutrition (Table S1).

### Effect of the investigated factors on Chl. a and carotenoids

**Fig. 2:**
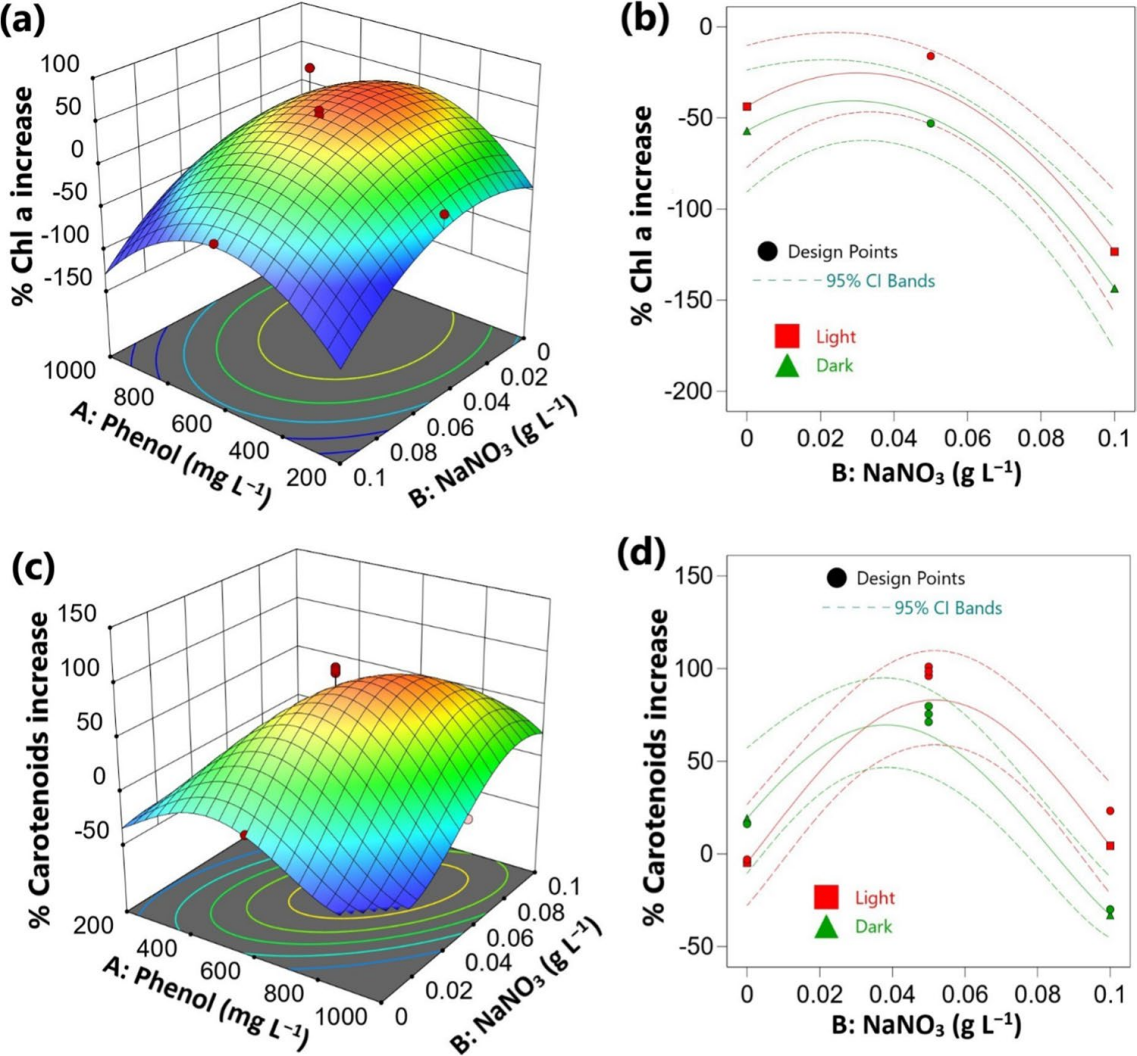
Effect of process variables on Chl a and carotenoids contents of *Chlorella* sp. In relation to the control cultures (**a**), (**c**) 3-D response surface plots under light conditions and (**b**), (**d**) interaction plot at 600 mg L^−1^ of phenol. CI: confidence interval

The Chl. a and carotenoid contents were calculated as mg g^−1^ DW and the results were expressed as a percentage increase in relation to the phenol-less controls (Table 1, 2). The highest Chl. a content of 11. 43 mg g^−1^ DW was observed at 600 mg L^−1^ of phenol and 0.05 g L^−1^ of NaNO_3_, which was promoted by 49.36% compared to the phenol-less control. While it was markedly reduced to 2.93 mg g^−1^ in the presence of 1000 mg L^−1^ of phenol (61.64% lower than the phenol-less control) under illuminated conditions (Table 1,2). The ANOVA analysis showed that the variations in phenol concentration had a non-significant effect on the percentage increase of Chl. a content in linear terms but showed strong significant negative effects in quadratic terms (Table S1). Accordingly, the percentage of Chl. a increase was relatively higher at moderate phenol concentrations compared to high phenol concentrations (1000 mg L^−1^) (Fig. 2a).

On the other hand, both NaNO_3_ concentration and nutritional mode showed significant negative effects on the percentage increase of Chl. a content (Table S1, Fig. 2b). In other words, the Chl. a contents were decreased by increasing nitrate concentrations, and this decrease was prominent under dark conditions. For instance, at 800 mg L^−1^ of phenol, the Chl. a content was reduced from 8.28 to 6.18 mg g^−1^ DW under illuminated conditions and from 6.25 to 4.42 mg g^−1^ DW in the absence of light when NaNO_3_ concentration was increased from 0.025 to 0.075 g L^−1^, respectively (Table 2).

In addition, non-significant mutual interaction between the investigated factors was observed regarding the percentage increase of Chl. a (Table S1). The highest increase of Chl. a contents was observed at 0.025 – 0.05 g L^−1^ of NaNO_3_ concentrations and 400 – 800 mg L^−1^ of phenol (Fig. 2a). At these concentrations, the percentage of Chl. a increase reached 49 – 74% in the mixotrophic cultures and 33 – 109% in the heterotrophic conditions compared to the phenol-less controls (Table 2).

On the other side, carotenoid contents were markedly increased under phenol treatments, thus increasing phenol concentration exhibited statistically significant positive effects on the percentage of carotenoids increase (Table S1). The highest content of carotenoids was observed in the treatment of 600 mg L^−1^ of phenol and 0.05 g L^−1^ of NaNO_3_, which reached 17.10 and 14.19 mg g^−1^ DW under mixotrophic and heterotrophic conditions, respectively (Table 2). This effect of phenol was enhanced by increasing NaNO_3_ concentrations. Therefore, phenol and nitrate exhibited a significant positive combined interaction on the percentage of carotenoids’ increase (Table S1, Fig. 2c). This result indicated that at higher NaNO_3_ (0.05 – 0.075 g L^−1^) and phenol concentrations (600 – 800 mg L^−1^), the carotenoids’ contents were highly stimulated to 51 – 98% under mixotrophic conditions, and 28 – 75% in heterotrophic cultures compared to the control (Table 2). However, this effect was reversed at elevated nitrate (0.1 g L^−1^) and phenol (800 – 1000 mg L^−1^) concentrations. This result was reflected by the significant negative quadratic effects of nitrate and phenol on the percentage of carotenoids’ increase (Table S1). In contrast, variations in nitrate concentration and culture condition showed non-significant effects in linear terms but significant negative mutual interaction (Table S1). This result indicated that the percentage of carotenoids’ increase had a remarkable variation between mixotrophic and heterotrophic modes at high nitrate concentrations (Table S1, Fig. 2d).

### Effect of the investigated factors on the cellular lipid contents

**Fig. 3:**
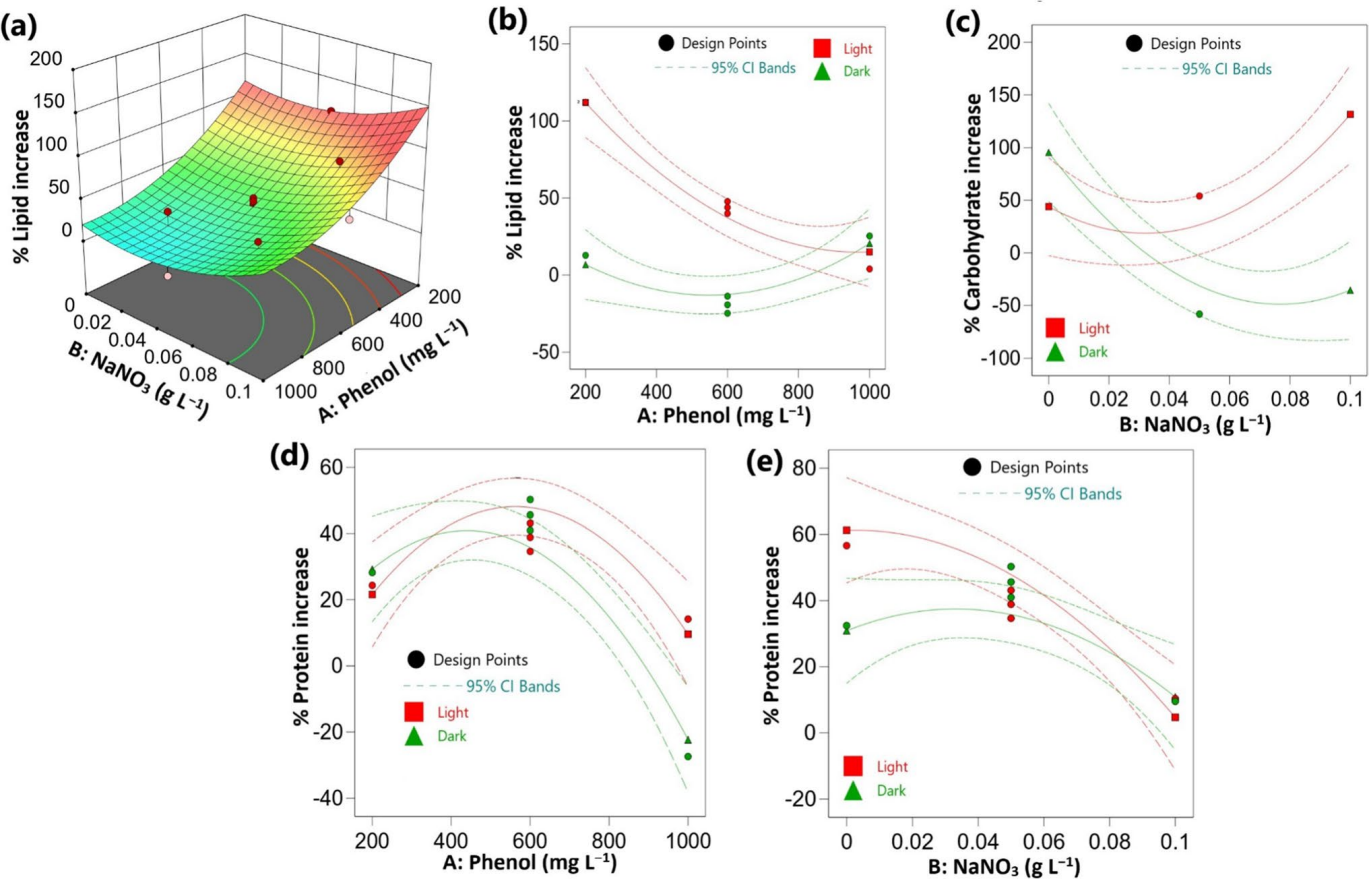
Effect of process variables on lipid, carbohydrate, and protein contents of *Chlorella* sp. in relation to the control (**a**) 3-D response surface plot under light conditions, (**b**), (**d**) interaction plots at 0.05 g L^−1^ of NaNO_3_ and (**c**), (**e**) interaction plots at 600 mg L^−1^ of phenol. CI: confidence interval

The three investigated factors showed high significant effects on the percentage increase of lipid contents, but their effects followed the following order: culture condition > phenol > NaNO_3_ (Table S1). Increasing nitrate concentrations exhibited positive effects, i.e. the percentage increase of lipid contents was higher at high NaNO_3_ concentrations than low concentrations (Fig. 3a). In contrast, in the control cultures, the highest lipid contents were observed under nitrogen-deplete conditions either in the presence of light (18.98% w/w) or dark conditions (22.73% w/w) (Table 1). However, the untreated cells tended to accumulate more lipids in the dark compared to the illuminated cells. In contrast, the cellular lipid contents under mixotrophic phenol treatments were relatively higher than the control cultures, thus, the percentage increase in lipid accumulation showed positive values and was fluctuated between 3.95 and 112.09% higher than the phenol-less controls (Table 3). The highest lipid content under mixotrophic phenol treatment was 26.26% w/w at 200 mg L^−1^ of phenol + 0.05 g L^−1^ of NaNO_3_, which corresponds to 12.38% w/w in the control (Table 1, 3). While under heterotrophic conditions in the presence of 600 mg L^−1^ of phenol + 0.0 g L^−1^ of NaNO_3_, the lipid content was 25% w/w, which corresponds to 22.73% w/w in the control (Table 1, 3). Accordingly, the presence of phenol induced an enhancing effect on algal lipid contents, but this effect was much pronounced under the mixotrophic phenol treatments. The ANOVA results supported these observations and indicated that the variation in phenol concentration and culture conditions had negative effects on the percentage of lipid increase (Table S1). Additionally, a significant positive interaction was observed between phenol and culture condition (Table S1). Therefore, the percentage of lipid increase was higher under mixotrophic treatments at low phenol concentrations (Fig. 3b).

**Table 3:** Effect of different of different phenol and sodium nitrate concentrations on carbohydrate, lipid and protein contents of *Chlorella* cells under light and dark condition

Factors				Responses					
No	Phenol (mg L^−1^)	NaNO_3_ (g L^−1^)	Culture condition	Carbohydrate content (%)	Carbohydrate increase (%)^a^	Lipid content (%)	Lipid increase (%)^a^	Protein content (%)	Protein increase (%)^a^
1	200	0.05	Light	29.67	54.04	26.26	112.09	13.32	24.36
2	400	0.025	Light	27.31	27.96	18.61	59.95	11.74	61.04
3	400	0.075	Light	40.78	97.71	23.31	89.57	13.78	28.21
4	600	0	Light	31.99	82.73	22.35	17.74	13.34	56.63
5	600	0.05	Light	34.37	78.43	17.81	43.87	18.59	38.86
6	600	0.1	Light	46.76	160.27	22.31	64.58	14.47	10.33
7	800	0.025	Light	33.36	56.33	15.54	33.62	11.59	58.98
8	800	0.075	Light	40.11	94.46	17.39	41.40	12.33	14.68
9	1000	0.05	Light	31.04	61.18	12.87	3.95	12.23	14.17
10	200	0.05	Dark	24.37	− 58.13	20.10	12.78	9.88	28.24
11	400	0.025	Dark	50.16	74.83	15.07	− 19.45	8.42	23.66
12	400	0.075	Dark	34.91	− 22.59	17.82	− 3.16	11.14	34.13
13	600	0	Dark	64.97	161.06	25.47	12.02	7.36	32.42
14	600	0.05	Dark	64.62	11.02	14.38	− 19.313	11.22	45.63
15	600	0.1	Dark	60.65	18.83	22.75	43.16	8.69	9.51
16	800	0.025	Dark	38.46	34.05	18.48	− 1.26	8.14	19.54
17	800	0.075	Dark	55.50	23.06	17.41	− 5.39	7.87	− 5.23
18	1000	0.05	Dark	40.56	− 30.31	22.34	25.38	5.60	− 27.37

^**a**^ % Increase = [(Treatment value – Control value)/ control value] × 100, values were calculated in relation to the corresponding control values listed in Table S1

### Effect of the investigated factors on carbohydrate contents

The results of the present study indicated that the contents of the intracellular soluble carbohydrates of *Chlorella* cells were higher in most of the treatments under heterotrophic phenol treatments than the mixotrophic conditions (Table 3). For instance, the microalgal cells were able to accumulate > 60% w/w of soluble carbohydrates at 600 mg L^−1^ of phenol under dark conditions, while in the presence of light, the contents were less than 47% w/w (Table 3). Furthermore, the soluble carbohydrate contents under mixotrophic phenol treatments were relatively higher than the control cultures, thus their percentage increase values were ranged between 27.96% at 400 mg L^−1^ of phenol + 0.025 g L^−1^ of NaNO_3_ and 160.27% at 600 mg L^−1^ of phenol + 0.1 g L^−1^ of NaNO_3_ (Table 3).

The variations in phenol and nitrate concentrations showed non-significant effects on the percentage increase of carbohydrate contents of the phenol-treated cultures compared to the phenol-less controls (Table S2). Conversely, the percentage of carbohydrate increase was significantly affected by the mode of nutrition which reflected higher values under mixotrophic condition compared to heterotrophic one. In addition, the mutual interaction between NaNO_3_ and culture condition also exhibited a significant negative effect on the percentage of carbohydrate increase (Table S2, Fig. 3c). Accordingly, at 600 mg L^−1^ of phenol, the increase in nitrate concentration from 0 to 0.1 g L^−1^ under illuminated conditions induced a marked increase in the soluble carbohydrates from 31.99 to 46.76% w/w (Table 3). While, in the absence of light, the soluble carbohydrates were decreased from 64.97 to 60.65% w/w when the NaNO_3_ concentration was increased from 0 to 0.1 g L^−1^.

### Effect of the investigated factors on protein contents

The total protein contents were generally higher under mixotrophic phenol treatment compared to the heterotrophic conditions, which contrasts the trend of soluble carbohydrates (Table 3). The ANOVA analysis indicated that the three investigated factors had significant negative effects on the percentage increase of total protein and the highest effects were attributed to NaNO_3_ concentration followed by phenol and culture condition (Table S2). In addition, phenol showed strong significant negative effects in quadratic terms, thus, the percentage of protein increase was promoted at phenol concentration up to 600 mg L^−1^ and 0.05 g L^−1^ of NaNO_3_ (18.59% w/w), and a marked decrease was observed above this concentration (Table 3, Fig. 3d). Furthermore, culture condition exhibited a significant positive mutual interaction with NaNO_3_ but a significant negative interaction with phenol (Table S2, Fig. 3e). Therefore, the protein contents were increased by increasing the NaNO_3_ from 0.025 to 0.075 g L^−1^ in most of the treatments.

In general, the total protein contents under mixotrophic phenol treatments were relatively higher than the control culture, thus an increase of 10.33% was observed at 600 mg L^−1^ of phenol + 0.1 g L^−1^ of NaNO_3_, and a maximum increase of 61.04% occurred at 400 mg L^−1^ of phenol + 0.025 g L^−1^ of NaNO_3_ (Table 3).

### Effect of the investigated factors on hydrogen peroxide (H_2_O_2_) contents

**Fig. 4:**
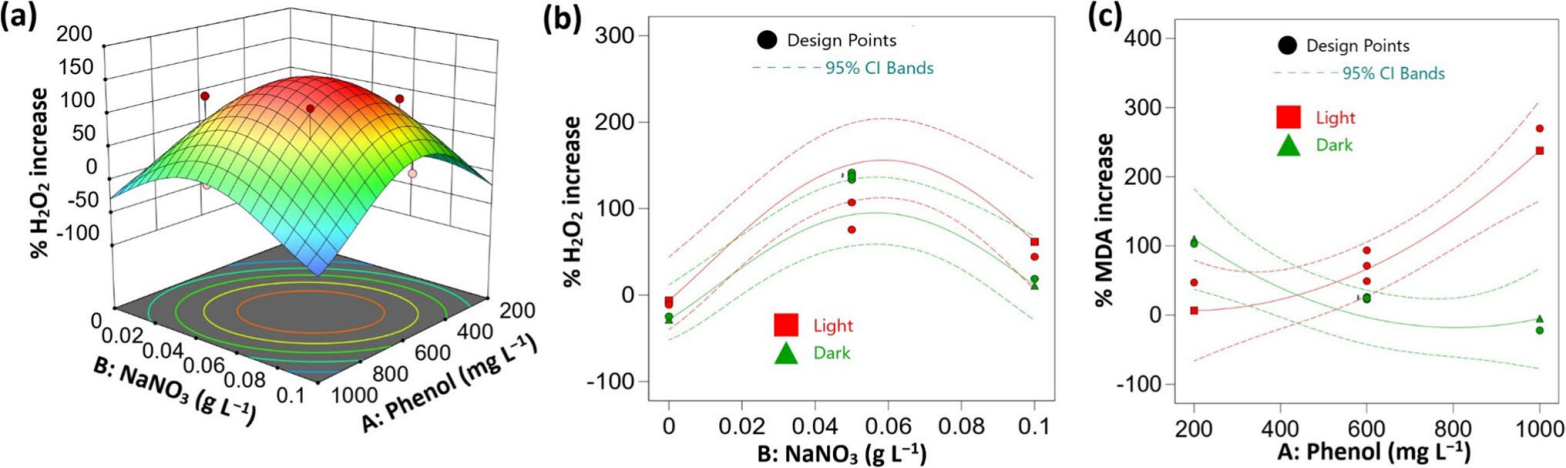
Effect of process variables on H_2_O_2_ and malonaldehyde (MDA) contents of *Chlorella* sp. in relation to the control (**a**) 3-D response surface plot under light conditions, (**b**) interaction plot at 600 mg L^−1^ of phenol and (**c**) interaction plot at 0.05 g L^−1^ of NaNO_3_. CI: confidence interval

ANOVA analysis showed that increasing the concentration of phenol and NaNO_3_ in the culture medium had statistically significant positive effects on the percentage of H_2_O_2_ increase (Table S2, Fig. 4a). Conversely, the mode of nutrition showed a significant negative effect, thus higher values of the percentage increase of H_2_O_2_ were observed under mixotrophic phenol treatment compared to heterotrophic ones (Fig. 4b). The mutual interaction between the three investigated factors exhibited non-significant effects on the percentage of H_2_O_2_ increase (Table S2). The highest values for the percentage increase of H_2_O_2_ were observed at 800 mg L^−1^ of phenol and 0.025 – 0.075 g L^−1^ of NaNO_3_ (Table 4, Fig. 4a). Accordingly, these factors showed strong significant negative quadratic effects on the percentage of H_2_O_2_ increase (Table S2).

**Table 4:** Effect of different of different phenol and sodium nitrate concentrations on antioxidant enzymes (catalase, CAT and ascorbate peroxidase, APX) and stress biomarkers (H_2_O_2_ and malonaldehyde, MDA) contents of *Chlorella* cells under light and dark condition

Factors				Responses							
No	Phenol (mg L^−1^)	NaNO_3_ (g L^−1^)	Culture condition	CAT (Umg^−1^)	CAT increase (%)^a^	APX (Umg^−1^)	APX increase (%)^a^	H_2_O_2_ (µmol g^−1^)	H_2_O_2_ increase (%)^a^	MDA (µmol g^−1^)	MDA increase (%)^a^
1	200	0.05	Light	2.19	− 55.14	0.68	− 67.96	8.92	2.70	21.02	47.14
2	400	0.025	Light	2.93	− 51.25	2.93	− 13.33	15.39	19.97	61.15	− 3.57
3	400	0.075	Light	2.13	− 72.75	1.14	− 43.69	8.36	115.69	27.71	− 30.42
4	600	0	Light	3.77	− 28.67	1.33	9.09	8.42	− 11.17	19.77	− 17.53
5	600	0.05	Light	6.15	25.96	2.25	5.60	17.99	107.05	24.48	71.34
6	600	0.1	Light	2.61	− 20.92	1.18	− 64.41	25.72	44.27	35.46	− 52.73
7	800	0.025	Light	4.32	− 28.13	3.00	− 11.11	28.84	124.73	92.17	45.33
8	800	0.075	Light	3.66	− 53.23	1.22	− 39.58	9.28	139.33	106.93	168.55
9	1000	0.05	Light	1.13	− 76.92	0.60	− 71.87	11.67	34.32	52.85	269.94
10	200	0.05	Dark	20.80	113.16	2.31	− 62.11	6.41	− 56.03	15.53	102.98
11	400	0.025	Dark	24.68	181.05	4.02	− 31.37	9.43	− 27.15	57.35	36.47
12	400	0.075	Dark	7.46	− 3.67	2.49	− 51.83	16.87	10.58	6.61	− 79.79
13	600	0	Dark	15.55	262.85	3.84	5.00	14.33	− 24.73	12.69	− 50.93
14	600	0.05	Dark	21.22	117.50	4.34	− 28.91	34.63	137.43	9.52	24.41
15	600	0.1	Dark	5.99	− 44.49	2.00	− 62.99	12.00	18.83	21.40	− 47.30
16	800	0.025	Dark	21.39	143.59	2.93	− 50.00	14.91	15.20	22.98	− 45.31
17	800	0.075	Dark	9.71	25.28	2.24	− 56.63	17.88	17.20	2.43	− 92.58
18	1000	0.05	Dark	5.17	− 47.06	1.15	− 81.18	16.14	10.68	5.96	− 22.03

^**a**^ % Increase = [(Treatment value – Control value)/ control value] × 100, values were calculated in relation to the corresponding control values listed in Table S1

### Effect of the investigated factors on Malonaldehyde (MDA) contents

The percentage of MDA increase was markedly higher under mixotrophic phenol treatments in relation to the heterotrophic conditions, thus culture condition exhibited high statistically significant negative effects. Furthermore, the effects of NaNO_3_ and phenol concentrations showed non-significant effects on the percentage increase of MDA. Regarding the interactive effects, the significant negative effect was observed for the mutual interaction between phenol concentration and culture conditions (Table S2). Thus, increasing phenol concentration enhanced the percentage of MDA increase in the presence of light, while the opposite effect was observed under dark conditions (Fig. 4c). In contrast, the mutual interaction between NaNO_3_ and culture condition as well as phenol and NaNO_3_ concentrations exhibited non-significant effects on the percentage of MDA increase (Table S2). Additionally, increasing NaNO_3_ concentration had statistically significant negative effects on the percentage of MDA increase at its quadratic term (Table S2). The highest values for MDA increase (269.94%) were observed under mixotrophic conditions at 1000 mg L^−1^ of phenol (Table 4).

### Effect of the investigated factors on antioxidant enzymes

**Fig. 5:**
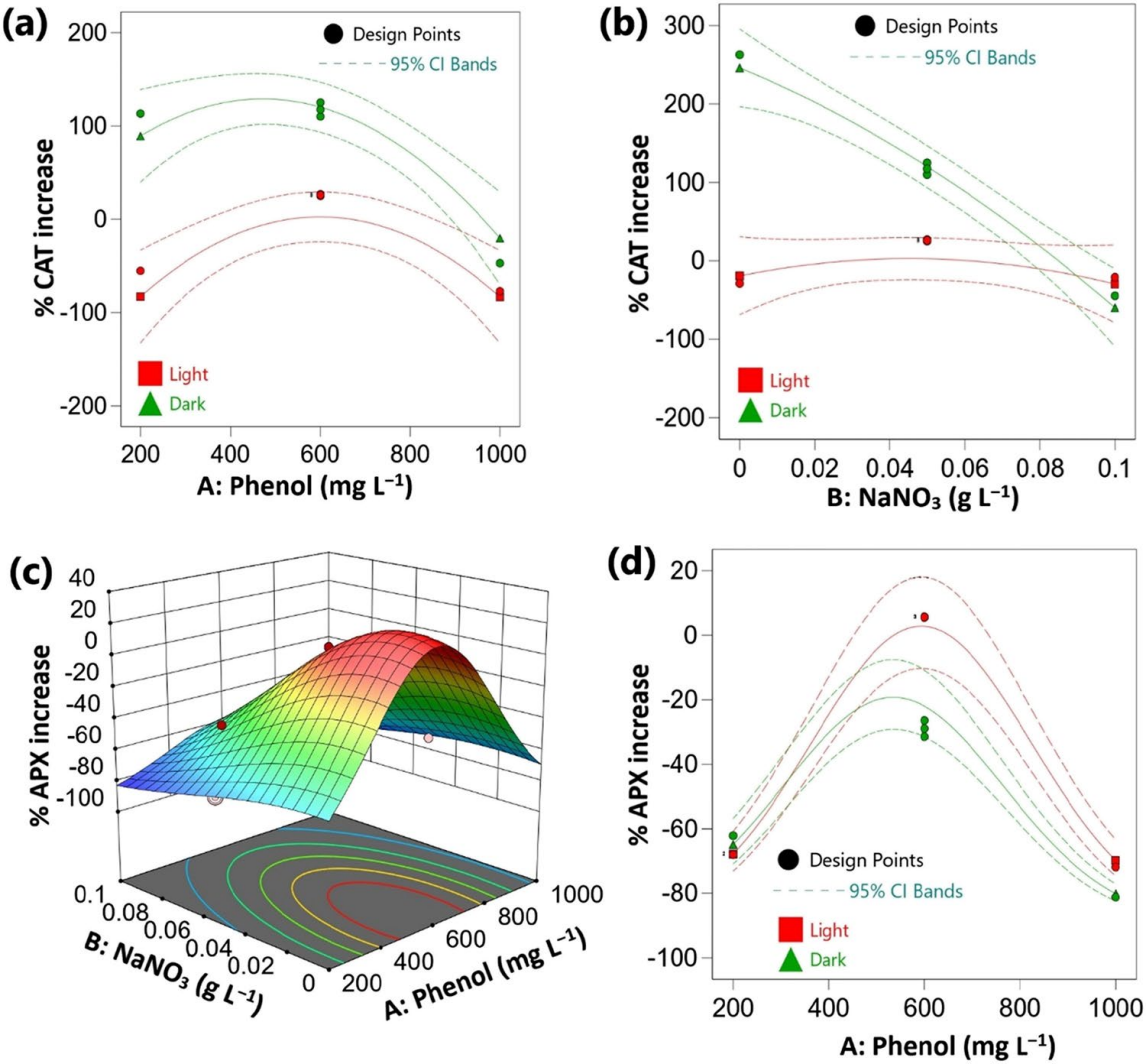
Effect of process variables on catalase (CAT) and ascorbate peroxidase (APX) contents of *Chlorella* sp. in relation to the control (**a**), (**d**) interaction plots at 0.05 g L^−1^ of NaNO_3_ (**b**) interaction plot at 600 mg L^−1^ of phenol and (**c**) 3-D response surface plot under light conditions. CI: confidence interval

The activities of catalase (CAT) and ascorbate peroxidase (APX) under different treatments were measured and expressed as U mg^−1^ protein and analyzed as percentage increase in relation to the phenol-less control (Table 4). The three studied factors exhibited statistically significant effects on CAT and APX activities of *Chlorella* cells. The importance of the investigated factors on the percentage increase of CAT activity followed the order: culture condition > NaNO_3_ > phenol concentration for CAT (Table S2). Conversely, in case of APX, the highest effect was related to NaNO_3_ followed by culture condition and phenol concentration (Table S2). Increasing the concentration of either phenol or NaNO_3_ in the cultivation medium had a negative effect on the percentage increase of both CAT and APX (Table S2, Fig. 5a-d). In contrast, the percentage of CAT increase was positively affected by culture condition (Table S2, Fig. 5a,b). Accordingly, CAT activity was relatively higher under heterotrophic condition compared to mixotrophic phenol treatments (Table 4, Fig. 5a,b). However, the opposite trend was observed in case of APX, culture condition showed negative effects, thus the percentage of APX increase were relatively higher in mixotrophic conditions (Table 4, Fig. 5c,d). Furthermore, the highest values for percentage increase of CAT and APX were observed at 600 mg L^−1^ of phenol. Accordingly, phenol concentration exhibited significant negative quadratic effects. On the other hand, culture condition exhibited significant negative mutual interactions with phenol on the percentage increase of CAT and APX (Table 2, Fig. 5a-d). Furthermore, the percentage of CAT increase was negatively influenced by the mutual interaction between culture condition and NaNO_3_ (Table S2).

## Discussion

Phenol is one of the most common organic pollutants in the aquatic environment which exerts several adverse effects on microalgae (El-Naeb et al. 2022). However, at non-lethal concentrations of phenol, microalgae can metabolize phenol as a source of organic carbon for growth and metabolism (Das et al. 2016; Zhang et al. 2020). Thus, in the presence of light, the algal growth becomes mixotrophic. While in the absence of light, photosynthesis cannot occur, but algal cells can still utilize phenol as a source of organic carbon, and the conditions become heterotrophic. In the present study, the phyco-toxicity of phenol towards *Chlorella* sp. was monitored under mixotrophic and heterotrophic conditions in the presence of different concentrations of phenol and NaNO_3_.

At low concentrations of phenol (< 400 mg L^−1^), a promoting effect on the microalgal biomass was observed, which was accompanied by high phenol removal efficiency. In contrast, high phenol concentrations (> 400 mg L^−1^) exhibited inhibitory effects on *Chlorella* growth. These results implied that phenol has a hormesis effect on microalgae, which is characterized by a low-dose stimulation and a high-dose inhibition. This phenomenon has been well documented in several organisms, including algae, for different toxic compounds (Kamaya et al. 2006). Similarly, Fawzy and Alharthi (2021) observed a hormesis effect of phenol on *Scenedesmus abundans*. Moreover, the growth inhibitory values observed in the present study did not exceed 50%. This implied that the effective phenol concentration that can cause 50% inhibition (EC_50_) on *Chlorella* cells was above 1000 mg L^−1^. In many previous studies, the EC_50_ of phenol towards microalgae was found to be 155 mg L^−1^ for *Dunaliella salina* (Cho et al. 2016), 74 mg L^−1^ for *Entomoneis* cf *punctulata* (Adams and Stauber 2004), 174 mg L^−1^ for *Pseudokirchneriella subcapitata* (Aruoja et al. 2011), 80 mg L^−1^ for *Microcystis aeruginosa*, 403 mg L^−1^ for *Scenedesmus quadricauda* and 631.4 mg L^−1^ for *Chlorella pyrenoidosa* (Tan et al. 2019). There are several biotic and abiotic factors that can affect the uptake and phyco-toxicity of phenol such as cell surface to volume ratio, variation in adaptation to toxic compounds, exposure time and environmental conditions (Newsted 2004; Cho et al. 2016). The present study indicated that nitrate concentration is an important parameter that stimulates effective uptake of phenol by *Chlorella* cells. This observation was consistent with Huang et al. (2022) who reported that the biodegradation of phenol by a bacterial strain is a nitrogen-dependent process. On the other hand, light was identified as an important limiting factor for the algal growth to mitigate the inhibitory effects of phenol, where the algal growth inhibition was relatively low in the absence of light. Newsted (2004) reported a marked variation in the algal growth under light and dark conditions, and these differences were related to phenol accumulation by the algal cells. The toxic effects of simple phenols is generally related to autoxidation processes (Nakai 2001), which are promoted by light and reflected by enhanced growth under mixotrophic conditions. Light can also play an important role in phenol degradation by microalgae, since photosynthesis can increase the oxygen levels in the medium, which promotes phenol oxidation (Di Caprio et al. 2018). However, no significant promotion in phenol removal was observed in the present study under illuminated conditions. Similarly, Di Caprio et al. (2018) indicated non obvious effects of light on the removal of phenolic compounds from olive mill wastewater (OMW). Conversely, Lindner and Pleissner (2021) reported a promotion of phenol removal from OMW by microalgae, which may be attributed to different metabolic pathways due to the use of glucose as a carbon source for microalgae growth.

Phenolic compounds, even at low concentrations, can influence the cell membrane functions as well as the metabolic activities of the algal cells (Ji et al. 2014). Consequently, the cellular contents of pigments, carbohydrates, lipids, proteins, and enzymes can be altered in response to phenol exposure. The data of the current study indicated that Chl. a biosynthesis was highly promoted at 0.025 – 0.05 g L^−1^ of NaNO_3_ and 400 – 800 mg L^−1^ of phenol in relation to the untreated cultures. Similarly, the highest stimulating effect on carotenoids’ contents was observed at phenol concentration of 600 – 800 mg L^−1^ and NaNO_3_ concentration of 0.05 – 0.075 g L^−1^. Generally, the increase in Chl. a and carotenoids’ contents in stressed microalgal cells can act as a protective mechanism against the accumulated phenol-induced phenoxy radicals (Cho et al. 2016). This increase in Chl. a contents upon phenol exposure agreed with previous studies (Cho et al. 2016; Fawzy and Alharthi 2021).

The total lipid, total protein and soluble carbohydrate contents of *Chlorella* cells were markedly increased under mixotrophic phenol treatments in relation to the autotrophic cultures, thus their percentage increase values were positive. However, the percentage increase of lipids was higher at low phenol concentrations, which was concomitant to high phenol removal efficiency. Phenol at sub-lethal concentrations can stimulate lipid accumulation in several microalgae (Das et al. 2015, 2016). On the other hand, the percentage relative increase in both total proteins and soluble carbohydrates under mixotrophic phenol stress were generally higher at 600 mg L^−1^ of phenol. This result implied that the products of phenol degradation can be utilized as a source of carbon for both growth and biosynthesis of lipids, proteins, and carbohydrates. Similarly, Ni et al. (2013) observed an increase in soluble sugars and proteins of the cyanobacterium *Microcystis aeruginosa* when exposed to different polyphenolic compounds such as gallic acid, catechol and pyrogallol. Moreover, some reports demonstrated that microalgal cells have a regulatory mechanism which can control the allocation of carbon to lipids and carbohydrates; there is a competition between lipid and starch biosynthesis (Park et al. 2014). Therefore, at 200 mg L^−1^ of phenol, the percentage increase of lipids, carbohydrates, and proteins were ~ 112, 54, and 24%, respectively. This observation suggested that most of the carbon produced from phenol degradation was allocated to lipid biosynthesis. In general, the enhancement of lipid contents under toxic phenol stress can be regarded as a detoxification mechanism, by sequestering lipophilic compounds and reduction of their bioavailability (Duan et al. 2017; Zhang et al. 2020). Moreover, the algal biomass produced from mixotrophic phenol degradation could be utilized as a feedstock for biodiesel and bioethanol production as well as an animal feed.

The importance of nitrate concentration in the culture medium was dependent on the presence or absence of phenol. Therefore, *Chlorella* cells cultivated in phenol-free medium tended to accumulate more lipids under nitrate-deplete conditions (18.98% w/w in light and 22.73% in dark). In contrast, the incorporation of nitrate in the phenol-treated cultures was important to induce high lipid accumulation of the stressed cells under the mixotrophic conditions. Thus, increasing NaNO_3_ concentration exhibited significant positive effects on the percentage of lipid increase. Furthermore, the simultaneous promotion in both phenol removal efficiency and lipid accumulation at nitrogen-replete conditions suggested that nitrate plays a crucial role in the biodegradation of phenol and its allocation to lipid biosynthesis. The optimum level of nitrates and its ratio to the dissolved organic matter play a crucial role in regulating the growth and biomass production (Ranadheer et al. 2019; Fawzy et al. 2022; Gomaa et al. 2022).

The effect of nitrate on total protein contents contradicts its effect on lipid contents. Thus, high nitrate concentration induced protein accumulation under photoautotrophic conditions, but exhibited significant negative effects on the percentage of protein increase of the phenol-treated cultures. Therefore, at nitrogen-deprived conditions, the incorporation of 600 mg L^−1^ of phenol under illuminated conditions induced a promotion in protein contents to 13.34% w/w, compared to 8.52% w/w in the control. These results further confirm that microalgae can utilize part of the organic carbon-derived from phenol degradation in the protein biosynthesis.

On the other hand, light is generally considered as a crucial factor that can influence algal metabolism. The cells of *Chlorella* sp. tended to accumulate more lipids and proteins and less soluble carbohydrates in the presence of light and phenol compared to the non-illuminated cultures. Conversely, Hodaifa et al. (2009) reported that the carbohydrate and protein contents of *Scenedesmus obliquus* cultivated on olive mill wastewater was not dependent on the received light.

The exposure of microalgal cells to environmental stresses can stimulate the generation of reactive oxygen species (ROS) such as H_2_O_2_, ^•^O_2_^−^ and ^•^OH. Hydrogen peroxide is generally long-lived and characterized by high membrane permeability leading to various toxic effects on the cellular organelles. Most of the treatments of phenol induced a marked increase in H_2_O_2_ levels compared to the non-stressed cells, which agreed with previous reports (Gomaa et al. 2021). On the other hand, the accumulation of ROS can induce lipid peroxidation resulting in the formation of malondialdehyde (MDA), thus it represents a good indication to the oxidative deterioration of cells. In general, in photosynthetic organisms, the levels of ROS are stimulated under illuminated conditions as a consequence of electron transport reactions (Shao et al. 2008). This result may explain the elevated concentrations of H_2_O_2_ and MDA under mixotrophic phenol stress.

Generally, H_2_O_2_ acts as a precursor for highly reactive ^•^OH, thus scavenging or destruction of H_2_O_2_ is a fundamental process in the living cells. Catalases and ascorbate peroxidases are important antioxidant enzymes which can catalyze the degradation of H_2_O_2_. The increase in the specific activity of CAT upon exposure to phenol stress has been observed in different microalgae (Gomaa et al. 2021). The data reported here suggested that most of the treatments under dark conditions were characterized by a marked enhancement in specific activity of CAT in relation to the untreated cells. However, under heterotrophic phenol stress, the percentage of CAT increase was relatively higher under nitrate-deprived or low nitrate concentration. It was also reported that algae grown in the dark had higher activity of CAT than in the light (Shao et al. 2008). The results also indicated that light dependent regulation of CAT activity may account for the higher concentration of H_2_O_2_ observed under mixotrophic phenol treatment. Furthermore, the specific activity of APX under phenol stress was relatively lower than the control cultures, thus the percentage increase of APX exhibited negative values in most of the treatments. This result may imply that CAT was responsible for the detoxification of H_2_O_2_ more than APX. In general, H_2_O_2_ signaling can induce the expression of specific genes which encode antioxidant-defense system in cells such as catalases and ascorbate peroxidases (Gomaa et al. 2021). Thus, the downregulation of the activity of the antioxidant enzymes in stressed cells is a necessity for the activation of those genes and further plays an important role in the acclimatization of the organism to oxidative stress (Shao et al. 2008). In the present study, this can be achieved by downregulating of APX activity. However, the decrease in APX activity may also be related to the overproduction of H_2_O_2_ or the effect of phenol on APX synthesis. Similarly, previous studies observed an increase in the specific activity of CAT with a concomitant reduction in APX in cells exposed to phenol stress (Ni et al. 2013; Mofeed and Abdel-aal 2015; Fawzy and Alharthi 2021).

## Conclusion

The present study demonstrated that phenol had a dose–response phenomenon (hormesis effect). Thereby, at low phenol concentrations, positive effects on the algal growth, and cellular metabolites was observed, while an inhibition of growth and cellular metabolism was induced by high concentrations. The response surface methodology directly visualized the effects of the process variables on the responses, which implied their feasibility in ecotoxicology. Nitrate-replete conditions were fundamental to minimize phenol stress and maximize its utilization by the microalgal cells as well as to promote lipid accumulation. This is important for environmental sustainability, i.e., for the treatment of phenolic wastewater and simultaneous biodiesel production from the produced biomass. Furthermore, mixotrophic phenol treatment is more advantageous regarding this aspect to maximize phenol removal and cellular lipid contents. However, the inhibitory effect of phenol on the algal growth was increased in the presence of light compared to the dark conditions. On the other hand, Chl. a, carotenoids, lipids, MDA and catalase showed to play a protective role against phenol-induced stress. In overall, the results of the present study highlighted the importance of understanding how several abiotic factors directly or indirectly affect the behavior of organic pollutants in the environment and their effects on microalgae as a primary producer and a vector for pollutants into higher trophic levels.

## Supplementary Information

Below is the link to the electronic supplementary material.

Supplementary file1 (PDF 487 KB)

## Data Availability

The datasets used and/or analyzed during the current study are available from the corresponding author on reasonable request.
