# Supplementary material for: Hormesis effects of phenol on growth and cellular metabolites of Chlorella sp. under different nutritional conditions using response surface methodology
Source: Environ Sci Pollut Res Int. 2023 Mar 17;30(19):56904–19. doi: 10.1007/s11356-023-26249-1 (PMC10121499; doi:10.1007/s11356-023-26249-1)
Supplement: Supplementary file 1 — Supplementary file1 (PDF 487 KB) [file 11356_2023_26249_MOESM1_ESM.pdf]

SUPPORTING INFORMATION

Phyco-toxicity of phenol to the green microalga *Chlorella* sp. under different nutritional conditions based on multiple physiological processes using response surface methodology

Mohamed Gomaa[1]*, Eman H. El-Naeb[1], Awatief F. Hifney[1], Mahmoud S. Adam[1], and Mustafa A. Fawzy[1,2],

[1] Botany & Microbiology Department, Faculty of Science, Assiut University, 71516, Assiut, Egypt

[2] Biology Department, Faculty of Science, Taif University, 21974, Taif, KSA

*** Corresponding author**

Dr. Mohamed Gomaa

Botany and Microbiology Department, Faculty of Science, Assiut University, 71516 Assiut, Egypt

e-mail: m_gomaa@aun.edu.eg

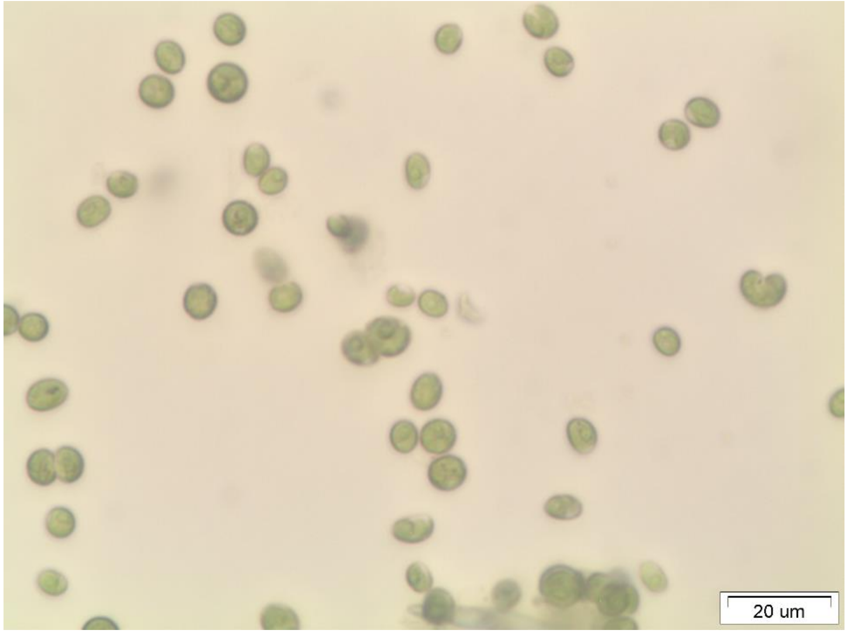

Fig. S1: Photomicrograph of *Chlorella* sp.

Table S1: Coefficient of estimates (CE) and the associated probability values (p−value) for each response variables of *Chlorella* sp.

**Factors**	**% Phenol removal**		**% Growth inhibition**		**% Chl a increase**		**% Carotenoids' increase** [a]		**% Lipid increase** [b]	
	CE	p−value	CE	p−value	CE	p−value	CE	p−value	CE	p−value
**Intercept**	29.58	< 0.0001	20.72	< 0.0001	38.93	< 0.0001	11.08	< 0.0001	12.29	< 0.0001
**A−Phenol**	−19.41	< 0.0001	16.42	< 0.0001	−1.45	0.64	1.05	0.001	−10.40	0.002
**B−$NaNO_3$**	3.80	0.0014	−2.01	0.079	−20.75	< 0.0001	−0.45	0.09	8.56	0.008
**C−Culture condition**	1.94	0.069	−5.55	0.0002	−8.39	0.019	−0.42	0.13	−24.93	< 0.0001
**AB**	−0.77	0.6434	−2.85	0.14	−3.05	0.57	1.48	0.004	−	−
**AC**	0.21	0.8288	−0.80	0.46	2.73	0.38	0.05	0.86	13.83	0.0002
**BC**	1.22	0.2145	−2.29	0.049	−0.84	0.78	−0.61	0.028	−	−
**A^2**	4.42	< 0.0001	−8.04	< 0.0001	−20.83	< 0.0001	−1.67	< 0.0001	6.57	0.009
**B^2**	−0.57	0.4544	2.26	0.018	−12.62	0.0001	−1.13	< 0.0001	5.52	0.024
**R^2**	0.975		0.970		0.913		0.903		0.899	

[a] This model was square root transformed as recommended by the Box-Cox analysis in the statistical software.

[b] This response was reduced based on backward elimination of insignificant factors.

R^2: Coefficient of determination

Table S2: Coefficient of estimates (CE) and the associated probability values (p−value) for each response variables of *Chlorella* sp.

**Factors**	**% Carbohydrate increase** [a]		**% Protein increase**		**% H_2O_2 increase** [b]		**% Malonaldehyde increase**		**% Catalase increase**		**% Ascorbate peroxidase increase** [c]	
	CE	p−value	CE	p−value	CE	p−value	CE	p−value	CE	p−value	CE	p−value
**Intercept**	43.78	< 0.0001	41.82	< 0.0001	13.4	0.0005	31.69	0.0005	61.34	< 0.0001	1.89	< 0.0001
**A−Phenol**	4.16	0.3219	-7.94	0.0012	1.02	0.012	14.54	0.12	-13.74	0.04	-0.06	0.0018
**B−$NaNO_3$**	-5.41	0.2026	-9.58	0.0003	0.82	0.036	-5.43	0.55	-39.53	< 0.0001	-0.12	< 0.0001
**C−Culture condition**	-28.91	< 0.0001	-6.05	0.0101	-1.09	0.01	-34.82	0.002	58.74	< 0.0001	-0.07	0.0008
**AB**	-	-	-5.84	0.1039	-0.78	0.22	27.38	0.096	7.85	0.46	0.02	0.53
**AC**	-	-	-4.95	0.0235	0.27	0.45	-43.25	0.0003	-13.67	0.04	-0.05	0.0067
**BC**	-27.33	< 0.0001	4.57	0.034	-0.11	0.76	-7.59	0.40	-36.94	< 0.0001	0.01	0.53
**A^2**	-9.45	0.01	-8.07	0.0001	-1.58	< 0.0001	13.92	0.07	-21.41	0.0006	-0.16	< 0.0001
**B^2**	15.31	0.0002	-3.73	0.029	-1.35	0.0003	-21.49	0.0085	-6.73	0.179	-0.05	0.0017
**R^2**	0.906		0.879		0.836		0.839		0.939		0.960	

[a] This response was reduced based on backward elimination of insignificant factors.

[b] This model was square-root transformed based on Box-Cox analysis in the statistical software.

[c] This model was Log transformed based on Box-Cox analysis in the statistical software.

R^2: Coefficient of determination
